# Supplementary material for: Multiplexed imaging mass cytometry reveals distinct tumor-immune microenvironments linked to immunotherapy responses in melanoma
Source: Commun Med (Lond). 2022 Oct 21;2:131. doi: 10.1038/s43856-022-00197-2 (PMC9587266; doi:10.1038/s43856-022-00197-2)
Supplement: Supplementary file 2 — Supplementary Information [file 43856_2022_197_MOESM2_ESM.pdf]

Supplementary Information

Multiplexed imaging mass cytometry reveals distinct tumor-immune microenvironments linked to immunotherapy responses in melanoma

Xu Xiao, Qian Guo, Chuanliang Cui, Yating Lin, Lei Zhang, Xin Ding, Qiyuan Li, Minshu Wang, Wenxian Yang, Yan Kong, Rongshan Yu

Supplementary Table 1: Patient clinicopathologic characteristics.

Characteristic	N = 26
Sex, N (%)	
Male	19 (73.1%)
Female	7 (26.9%)
Age/Years, N (%)	
≥ 60	9 (34.6%)
≤ 60	17 (65.4%)
median (range)	57 (30-72)
Race, N (%)	
Asian	26 (100%)
Tumor site, N (%)	
Acral	11 (42.3%)
Mucosal	6 (23.1%)
Cutaneous	5 (19.2%)
Unknown	4 (15.4%)
Tumor thickness, N (%)	
≤ 4 mm	2 (7.7%)
>4 mm	9 (34.6%)
Unknown	15 (57.7%)
Ulceration	
With	9 (34.6%)
Without	4 (15.4%)
Unknown	13 (50%)
TNM stage, N (%)	
I	1 (3.8%)
II	3 (11.5%)
III	6 (23.1%)
IV	16 (61.5%)
Efficacy, N (%)	
CR	1 (3.8%)
PR	10 (38.5%)
SD	3 (11.5%)
PD	12 (46.2%)

Supplementary Table 2: Patient clinical information.

ID	Sex	Age	Response	Tumor thickness	TNM	Site	IM ROI (N)	CT ROI (N)	isProgressed	PFS (months)	isDeceased	OS (months)
66	F	40-49	PR	Unknown	IV	Mucosal	1	1	1	13.57	0	39.27
87	F	60-69	SD	>4mm	IV	Mucosal	1	2	1	26.57	0	33.67
77	F	30-39	PD	1-2mm	IV	Acral	1	1	1	0.37	0	5.77
12	F	50-59	PD	Unknown	II/III	Acral	3	1	1	3.63	1	31.77
59	F	50-59	PD	Unknown	IV	Mucosal		2	0	2.33	1	8.63
73	F	60-69	SD	Unknown	IV	Acral	2	3	0	34.23	0	34.23
60	F	60-69	PR(4)	>4mm	IV	Cutaneous	1	2	0	56.23	0	56.23
64	F	60-69	SD	Unknown	III	Acral	2	1	1	25.50	0	43.40
84	F	70-79	PD	Unknown	IV	Unknown		3	1	2.80	0	8.03
85	F	50-59	PR	Unknown	IV	Acral	8	4	0	31.23	0	31.23
36	F	50-59	PR(2)	>4mm	III	Cutaneous	4	3	1	7.77	0	44.77
9	M	30-39	PD	2-4mm	II/III	Unknown	2	2	1	1.47	0	9.03
8	M	50-59	PD	>4mm	III	Cutaneous	2	1	1	4.50	1	15.60
33	F	60-69	PD	>4mm	IV	Mucosal	8	4	1	3.77	1	7.50
41	F	60-69	PD	Unknown	III	Cutaneous	6	2	1	3.27	0	45.37
38	M	50-59	PD	>4mm	IV	Cutaneous	3	2	1	4.47	0	10.77
45	F	40-49	CR(2)	Unknown	IV	Unknown	6	2	1	49.97	0	49.97
76	F	40-49	PD	Unknown	IV	Acral	8	3	1	2.40	1	5.80
79	M	30-39	PR	Unknown	IV	Mucosal	9	2	1	32.00	0	32.00
63	M	30-39	PR	>4mm	IV	Acral	4	3	0	19.43	0	45.03
67	F	50-59	PR	Unknown	I	Mucosal	6	3	0	49.47	0	49.47
52	M	60-69	PR(2)	Unknown	IV	Unknown	3	2	1	11.57	0	31.93
53	F	60-69	PD	>4mm	II	Acral	3	1	1	1.80	1	31.43
55	F	30-39	PD	Unknown	III	Acral	5	3	1	5.13	0	21.77
65	F	40-49	PR	>4mm	III	Acral	8	4	1	25.80	0	57.93
68	M	50-59	PR	Unknown	IV	Acral	3	2	1	29.90	0	32.17

Supplementary Table 3: Pathological response for all patients with different tumor site and stage.

	Overall, N (%)	Responder, N (%, 95% CI)[1]	Nonresponder, N (%, 95% CI)[1]	*P* value[2]
Tumor site				0.94
Acral	11 (42.3%)	6, (54.5%, 23-83)	5, (45.5%, 17-77)	
Mucosal	6 (23.1%)	4, (66.7%, 22-96)	2, (33.3%, 4-78)	
Cutaneous	5 (19.2%)	2, (40%, 5-85)	3, (60%, 15-95)	
Unknown	4 (15.4%)	2, (50%, 7-93)	2, (50%, 7-93)	
Tumor stage				0.16
I	1 (3.8%)	1 (100%, 5-100)	0 (0, 0-95)	
II	3 (11.5%)	0 (0, 0-63)	3 (100%, 37-100)	
III	6 (23.1%)	3 (50%, 12-88)	3 (50%, 12-88)	
IV	16 (61.5%)	10 (62.5%, 35-85)	6 (37.5%)	

[1] 95% CI are measured by two-sided Clopper-Pearson exact method

[2] *P* values are based on Fisher's exact tests to test association between response and tumor type.

Supplementary Table 4: Antibodies and concentrations.

Metal Tag	Targets	Clone	Dilution	Vendor
141Pr	CD38	EPR4106	1:100	Fluidigm
142Nd	PDGFRb	Y92	1:100	Abcam (ab271835)
143Nd	Vimentin	D21H3	1:100	Fluidigm
144Nd	CD14	EPR3653	1:100	Fluidigm
145Nd	CD127	EPR2955(2)	1:100	Abcam (ab240225)
146Nd	CD16	EPR16784	1:100	Fluidigm
147Sm	IDO	EPR20374	1:100	Abcam (ab271990)
148Nd	CD278 (ICOS)	D1K2T	1:100	Fluidigm
149Sm	CD194 (CCR4)	L291H4	1:100	Fluidigm
150Nd	CD274 (PD-L1)	E1L3N	1:100	Fluidigm
151Eu	CD134 (OX40)	Polyclonal	1:100	Fluidigm
152Sm	CD45	CD45-2B11	1:75	Fluidigm
153Eu	CD223 (LAG3)	D2G40	1:50	Fluidigm
154Sm	CD11c	Polyclonal	1:100	Fluidigm
155Gd	FOXP3	236A/E7	1:100	Fluidigm
156Gd	CD4	EPR6855	1:100	Fluidigm
158Gd	E-cadherin	24E10	1:100	Fluidigm
159Tb	CD68	KP1	1:200	Fluidigm
160Gd	EpCAM	EPR20532-222	1:50	Abcam (ab232539)
161Dy	CD20	H1	1:100	Fluidigm
162Dy	CD8a	C8/144B	1:100	Fluidigm
163Dy	VEGF	G153-694	1:75	Fluidigm
164Dy	CAIX	EPR23055-5	1:75	Abcam (ab270401)
165Ho	CD279 (PD-1)	EPR4877(2)	1:100	Fluidigm
166Er	CD74	LN2	1:100	Fluidigm
167Er	CD366 (TIM-3)	EPR22241	1:100	Abcam (ab242080)
168Er	Ki-67	B56	1:100	Fluidigm
169Tm	Collagen type I	Polyclonal	1:150	Fluidigm
170Er	CD3	Polyclonal	1:100	Fluidigm
171Yb	CD27	EPR8569	1:75	Fluidigm
172Yb	FAP	Polyclonal	1:100	Abcam (ab53066)
173Yb	CD11b	SP330	1:100	Abcam (ab241408)
174Yb	HLA-DR	LN3	1:100	Fluidigm
175Lu	Alpha-SMA	Polyclonal	1:200	Abcam (ab5694)
176Yb	AXL	EPR19880	1:100	Abcam (ab240396)
191Ir	DNA1			Fluidigm
193Ir	DNA2			Fluidigm

Supplementary Table 5: Melanoma RNA-seq cohorts used in this study.

	PUCH [1]	Riaz17 [2]	Gide19[3]	Liu19 [4]
Cohort size, N	55	51	50	54
Nonresponse	35	25	20	20
Response	14	10	23	28
Stable disease	6	16	7	6

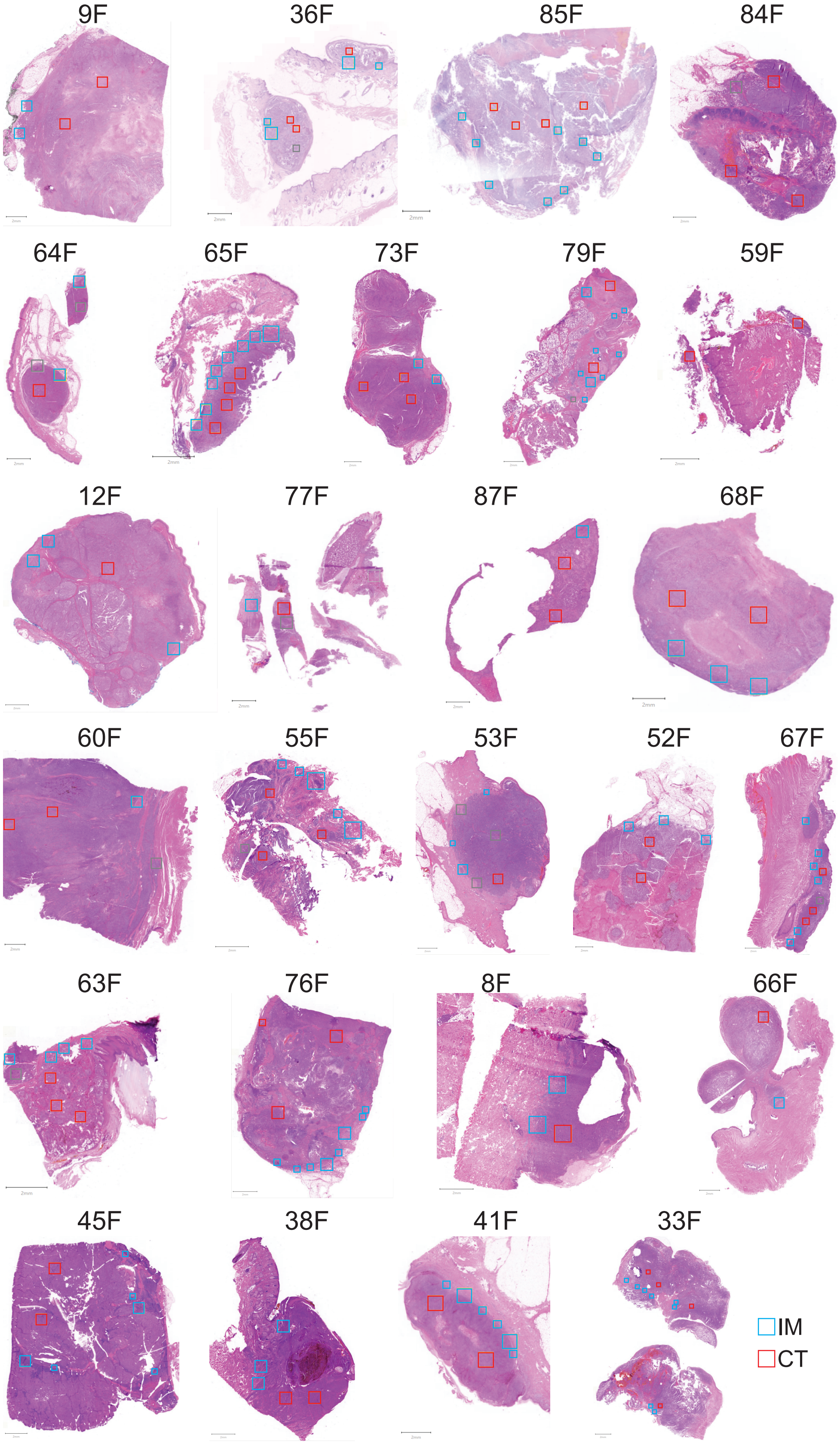

Supplementary Figure 1: Hematoxylin and eosin (HE)-stained images of all samples. Selected regions of interest (ROIs) for each sample are marked in the HE image. Blue boxes denote ROIs from invasive margin (IM) regions and red boxes denote ROIs from core tumor (CT) regions.

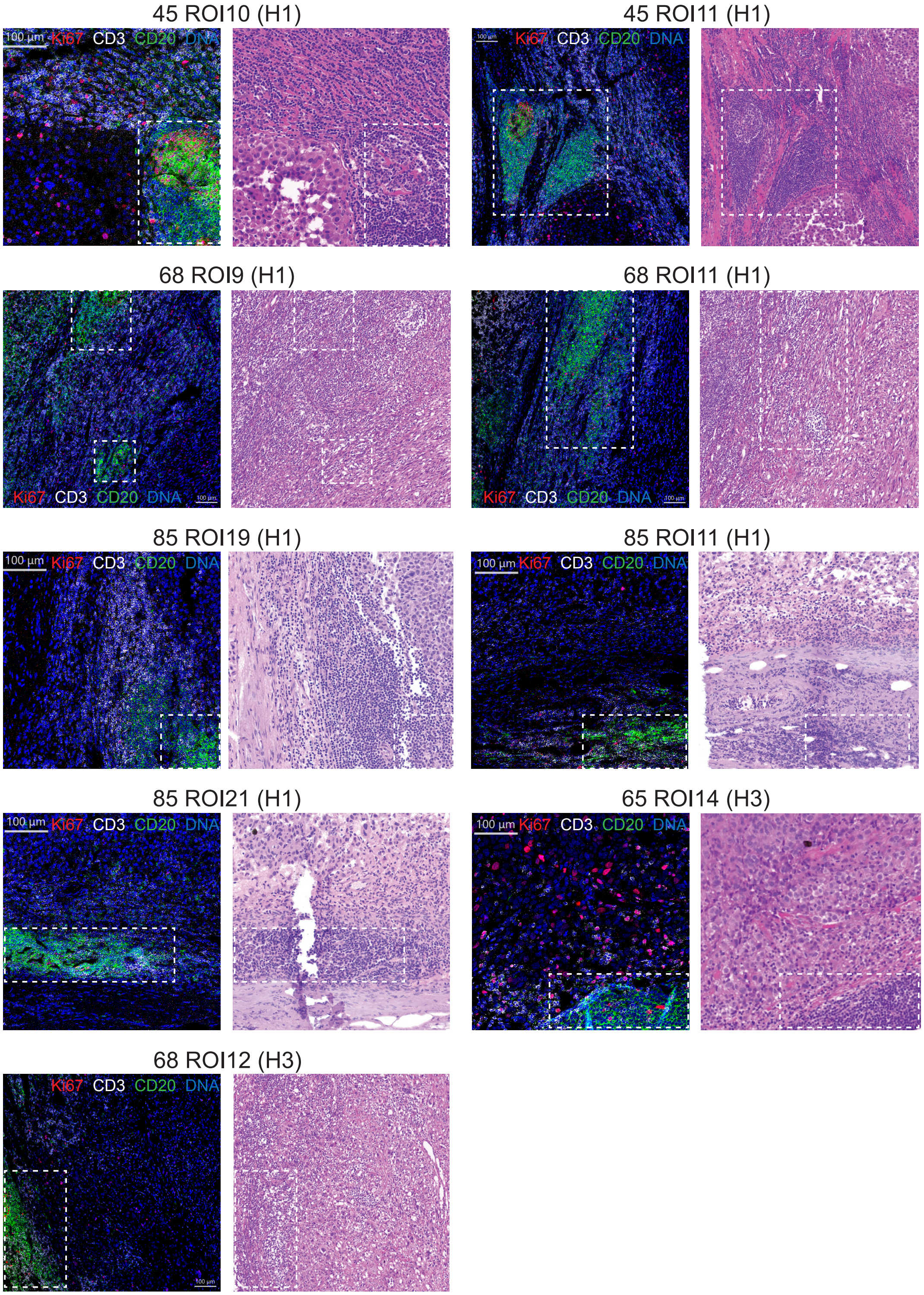

Supplementary Figure 2: Imaging mass cytometry (IMC) images with tertiary lymphoid structure (TLS) and the corresponding hematoxylin and eosin (HE)-stained images. The sample name with the region of interest (ROI) number and the tumor microenvironment type (inside the bracket) of the IMC image are on the top of each paired images (left: IMC image, right: HE image). The dotted boxes denote TLS regions determined by pathologist.

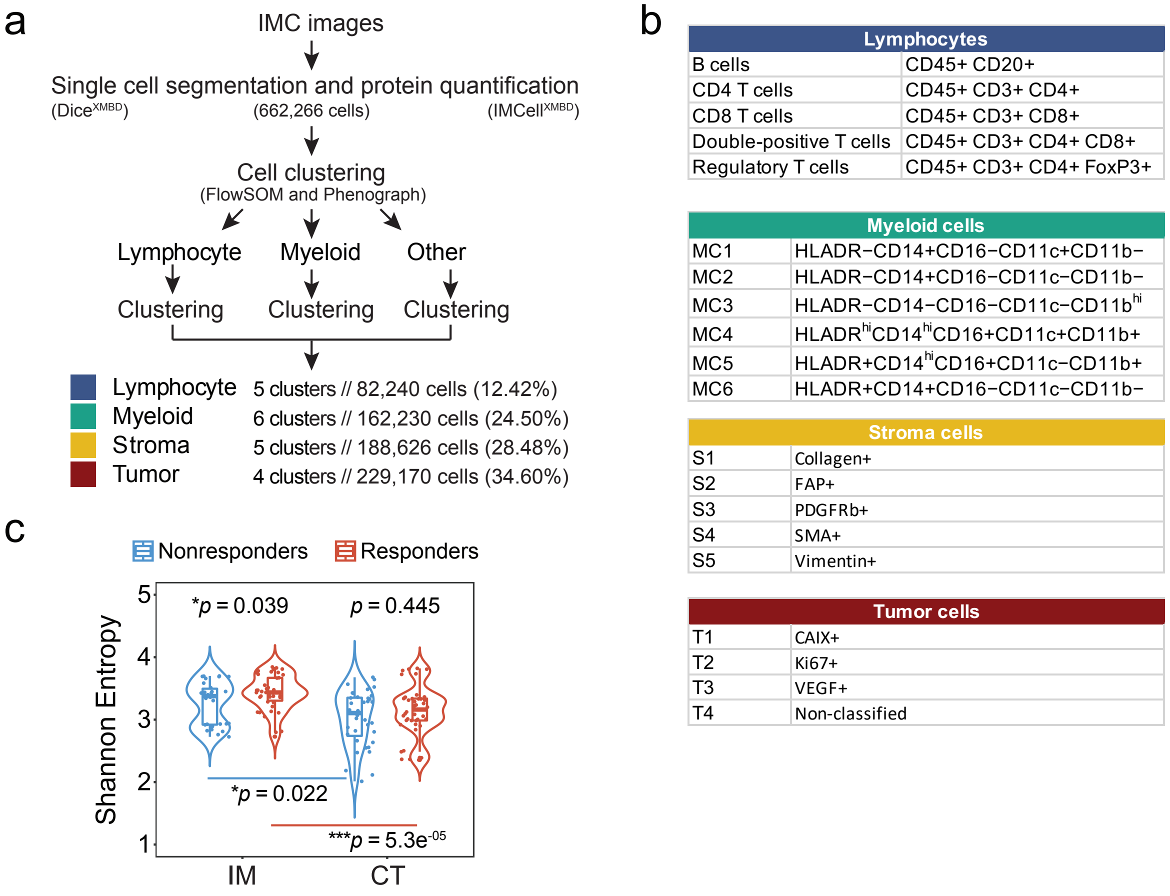

Supplementary Figure 3: The high-dimensional single cells phenotyping. **(a)** Flowchart detailing the preprocessing and analytical steps taken at iterative subsets of the single-cell cohort. **(b)** Four main cell types and 20 subtypes with representing markers. **(c)** Violin plots showing the distribution of intra-patient tumor heterogeneity measured by Shannon entropy for all samples (n = 42 for the R group and n = 30 for the NR group in the IM, n = 42 for R group and n = 36 for NR group in the CT). Boxplots inside violin plots are shown with the median (the center line), interquartile range (IQR), and 1.5 times the IQR (whiskers), with outliers exceeding 1.5 times the IQR. (Wilcoxon rank sum test, $*p < 0.05$, $***p < 0.001$). R: responder, NR: nonresponder, IM: invasive margin, CT: core tumor.

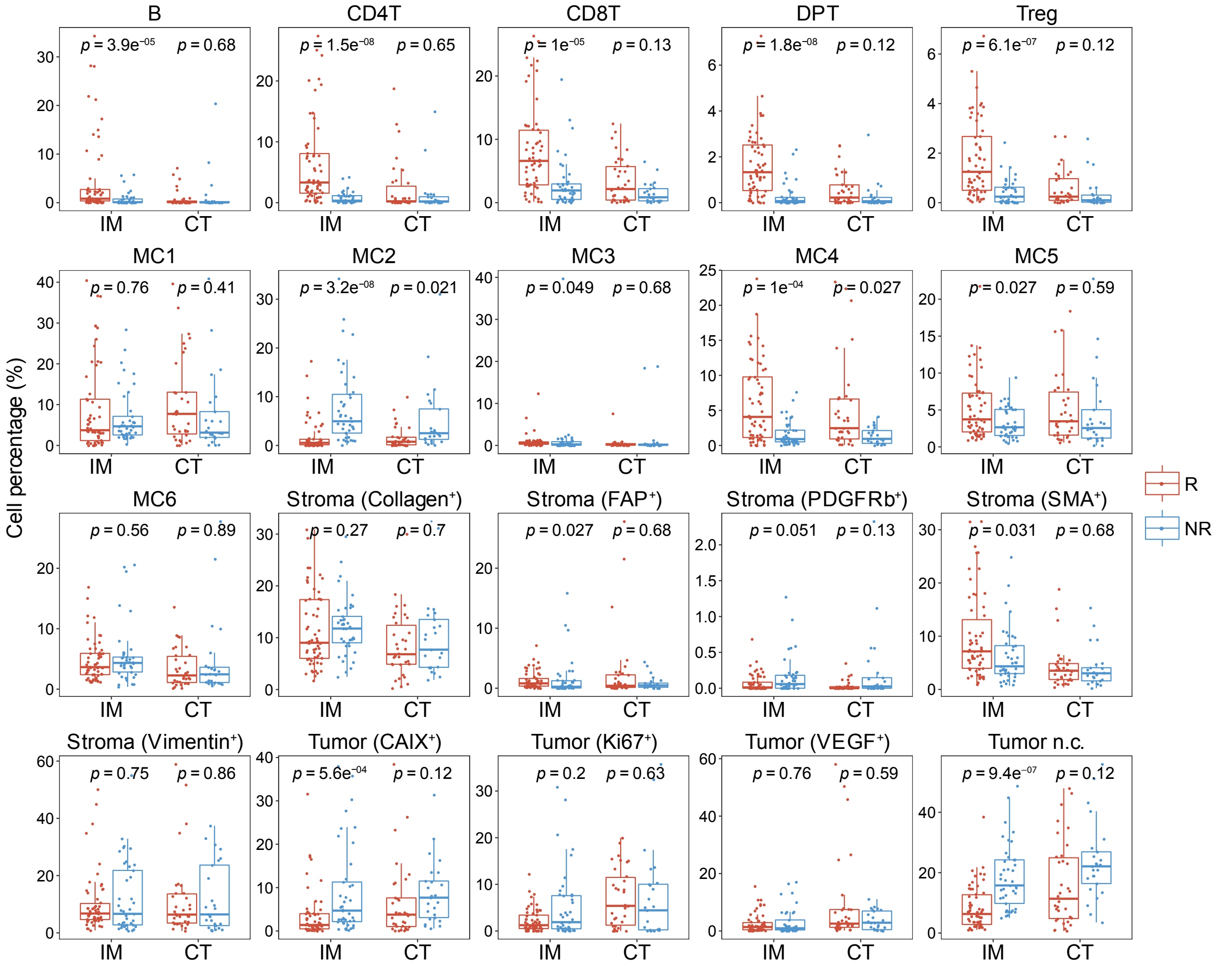

Supplementary Figure 4: The cell composition difference between responders and nonresponders. Boxplots showing the proportion of each cell type in responders (R, red) and nonresponders (NR, blue). Each boxplot is shown with the median (the center line), interquartile range (IQR), and 1.5 times the IQR (whiskers), with outliers exceeding 1.5 times the IQR. Points in the boxplot represent the cell percentage of each IMC image (n = 58 for R group and n = 41 for NR group in the IM, n = 34 for R group and n = 25 for NR group in the CT). Comparisons were performed using Wilcoxon rank sum test and adjusted with Benjamini-Hochberg method. IM: invasive margin, CT: core tumor.

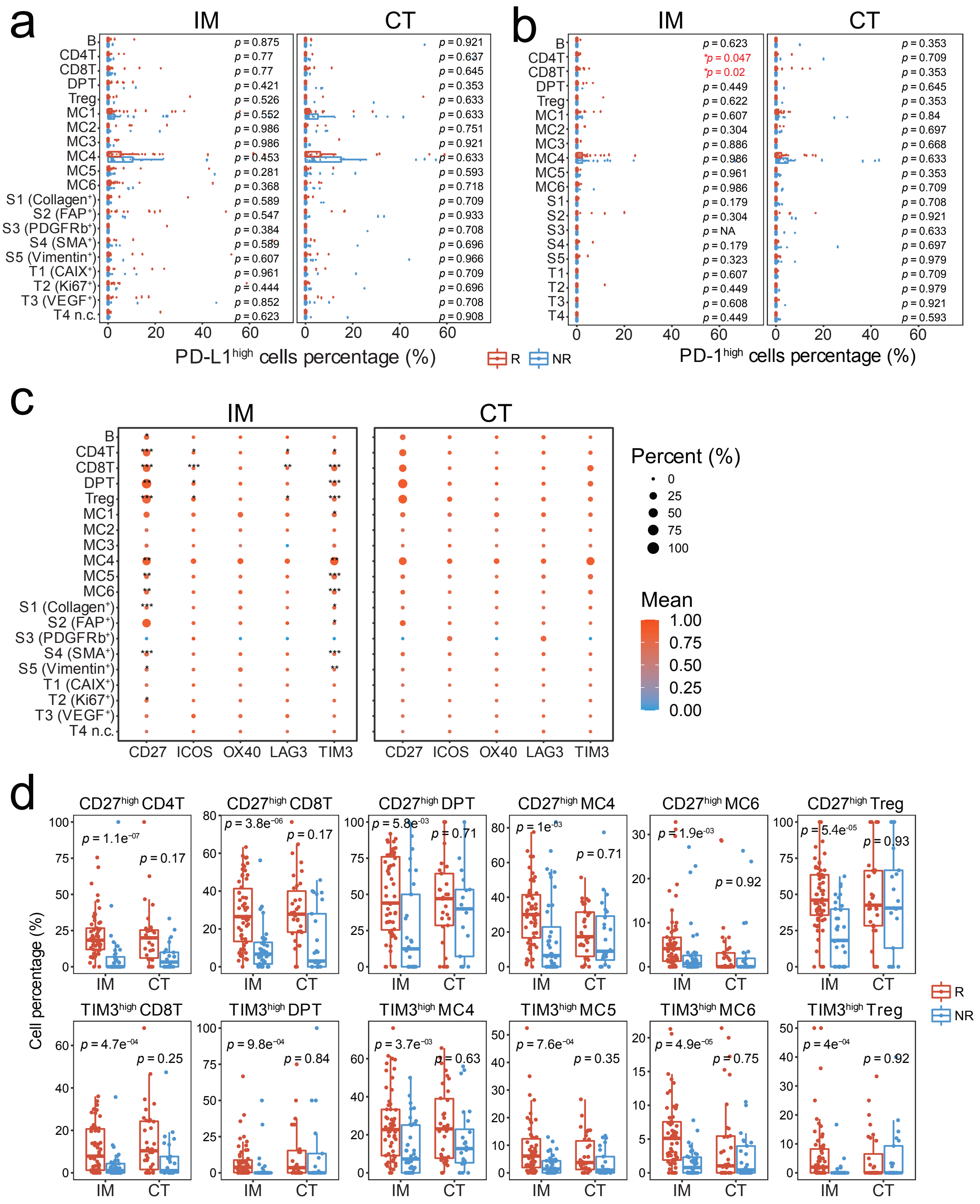

a
IM
CT
B
CD4T
CD8T
DPT
Treg
MC1
MC2
MC3
MC4
MC5
MC6
S1 (Collagen+)
S2 (FAP+)
S3 (PDGFRb+)
S4 (SMA+)
S5 (Vimentin+)
T1 (CAIX+)
T2 (Ki67+)
T3 (VEGF+)
T4 n.c.
p = 0.875
p = 0.77
p = 0.77
p = 0.421
p = 0.526
p = 0.552
p = 0.986
p = 0.986
p = 0.453
p = 0.281
p = 0.368
p = 0.589
p = 0.547
p = 0.384
p = 0.589
p = 0.607
p = 0.961
p = 0.444
p = 0.852
p = 0.623
p = 0.921
p = 0.637
p = 0.645
p = 0.353
p = 0.633
p = 0.633
p = 0.751
p = 0.921
p = 0.633
p = 0.593
p = 0.718
p = 0.709
p = 0.933
p = 0.708
p = 0.696
p = 0.966
p = 0.709
p = 0.696
p = 0.708
p = 0.908
0
20
40
60
PD-L1high cells percentage (%)
R
NR
b
IM
CT
B
CD4T
CD8T
DPT
Treg
MC1
MC2
MC3
MC4
MC5
MC6
S1
S2
S3
S4
S5
T1
T2
T3
T4
p = 0.623
*p = 0.047
*p = 0.02
p = 0.449
p = 0.622
p = 0.607
p = 0.304
p = 0.886
p = 0.986
p = 0.961
p = 0.986
p = 0.179
p = 0.304
p = NA
p = 0.179
p = 0.323
p = 0.607
p = 0.449
p = 0.608
p = 0.449
p = 0.353
p = 0.709
p = 0.353
p = 0.645
p = 0.353
p = 0.84
p = 0.697
p = 0.668
p = 0.633
p = 0.353
p = 0.709
p = 0.708
p = 0.921
p = 0.633
p = 0.697
p = 0.979
p = 0.709
p = 0.979
p = 0.921
p = 0.593
0
20
40
60
PD-1high cells percentage (%)
c
IM
CT
Percent (%)
0
25
50
75
100
Mean
1.00
0.75
0.50
0.25
0.00
CD27
ICOS
OX40
LAG3
TIM3
d
CD27high CD4T
p = 1.1e-07
p = 0.17
CD27high CD8T
p = 3.8e-06
p = 0.17
CD27high DPT
p = 5.8e-03
p = 0.71
CD27high MC4
p = 1e-03
p = 0.71
CD27high MC6
p = 1.9e-03
p = 0.92
CD27high Treg
p = 5.4e-05
p = 0.93
TIM3high CD8T
p = 4.7e-04
p = 0.25
TIM3high DPT
p = 9.8e-04
p = 0.84
TIM3high MC4
p = 3.7e-03
p = 0.63
TIM3high MC5
p = 7.6e-04
p = 0.35
TIM3high MC6
p = 4.9e-05
p = 0.75
TIM3high Treg
p = 4e-04
p = 0.92
Cell percentage (%)
IM
CT
R
NR

Supplementary Figure 5: The relationship between clinical response and expression level of checkpoint proteins per cell type. The expression level was the relative abundance with respect to the total number of corresponding cell types. Positive cells were defined with expression values over three quarters of all cells. **(a-b)** Boxplots showing the relative percentage of PD-L1 **(a)** and PD-1 **(b)** positive cells in samples from responders (R, red) and nonresponders (NR, blue). **(c)** Dotplots displaying the relative proportion of positive cells using dot size, the mean expression level of selected proteins based on scaled expression value. **(d)** Boxplots showing the relative abundance of CD27/TIM3 positive cells in samples from R (red) and nonresponders (NR, blue). Each boxplot is shown with the median (the center line), interquartile range (IQR), and 1.5 times the IQR (whiskers), with outliers exceeding 1.5 times the IQR. Points in the boxplot represent the cell percentage of each image. Comparisons between R and NR groups were performed using Wilcoxon rank sum test and adjusted with Benjamini-Hochberg method. *$p < 0.05$, **$p < 0.01$, ***$p < 0.001$. For all panels, n = 58 for R group and n = 41 for NR group in the invasive margin (IM), n = 34 for R group and n = 25 for NR group in the core tumor (CT).

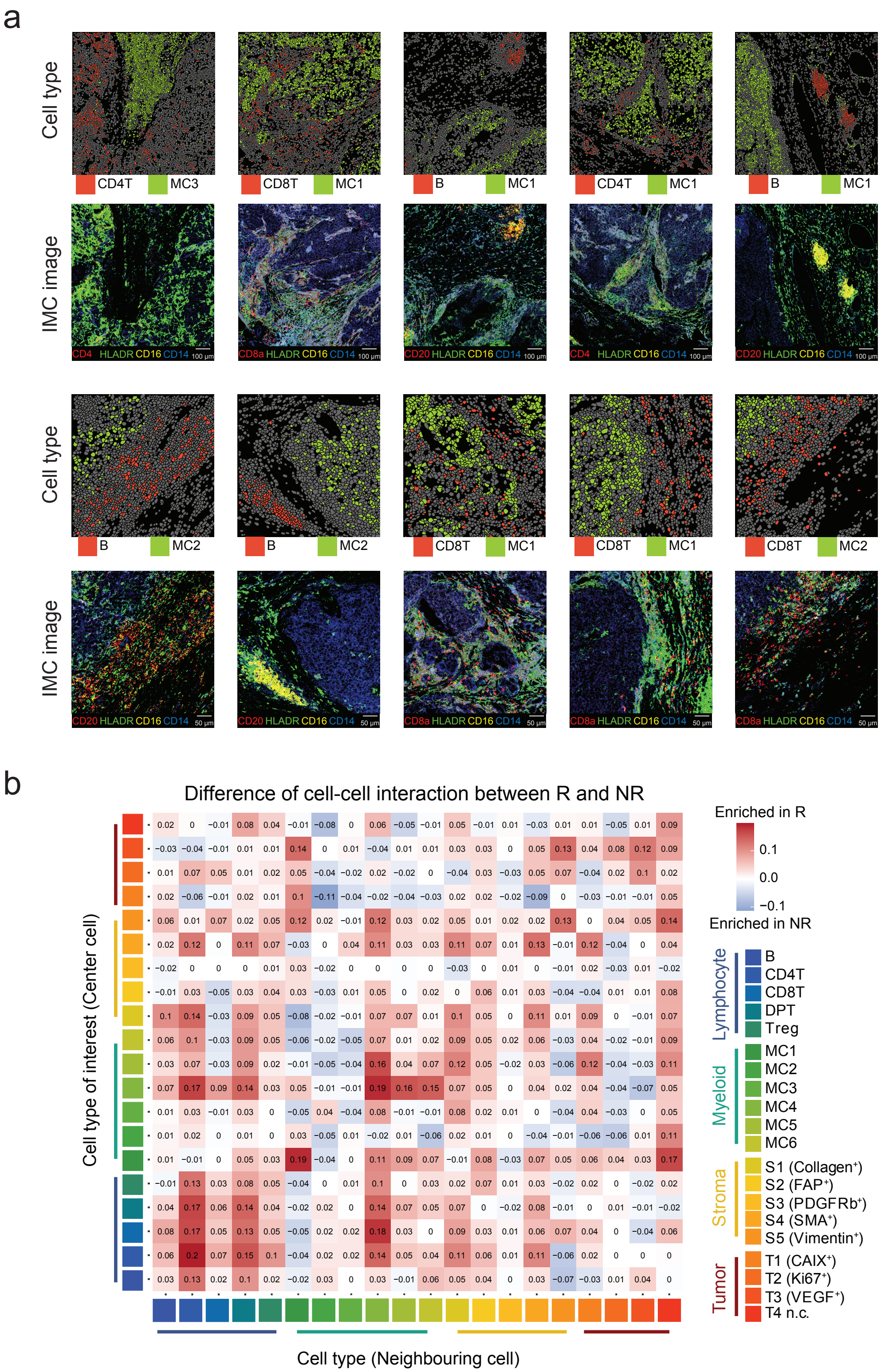

Supplementary Figure 6: Spatial analysis among cell phenotypes. **(a)** Representative imaging mass cytometry (IMC) images colored by cell type (the first/third rows) and marker (the second/fourth rows) showing cell-cell avoidance. The scale bar of each image is shown in the right bottom of the corresponding IMC image. **(b)** Heatmap displaying frequencies of significant images determined by permutation test with $P < 0.01$ between responders (R, n = 92) and nonresponders (NR, n = 66). Red color indicating interactions that are more frequent in the R group, and blue color indicating interactions that are more frequent in the NR group.

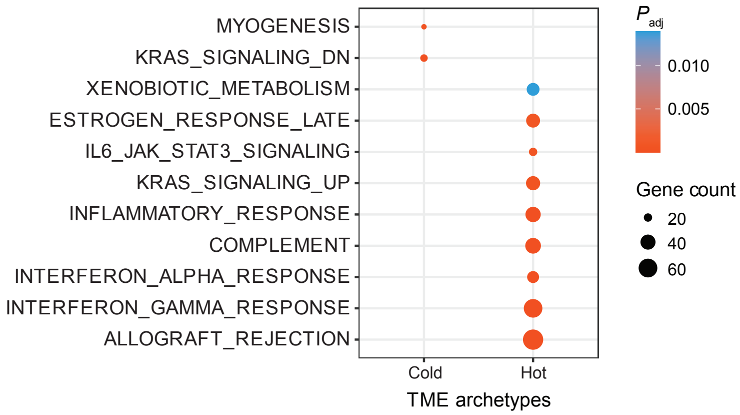

Supplementary Figure 7: Pathway analysis. Gene set enrichment analysis (GSEA) of genes up-regulated expressed in patients with immune cold (n = 13) or immune hot (n = 11) tumor microenvironment archetype. Significantly enriched gene sets (adjusted $P < 0.05$, Benjamini-Hochberg method) from MSigDB HALLMARK collection are shown.

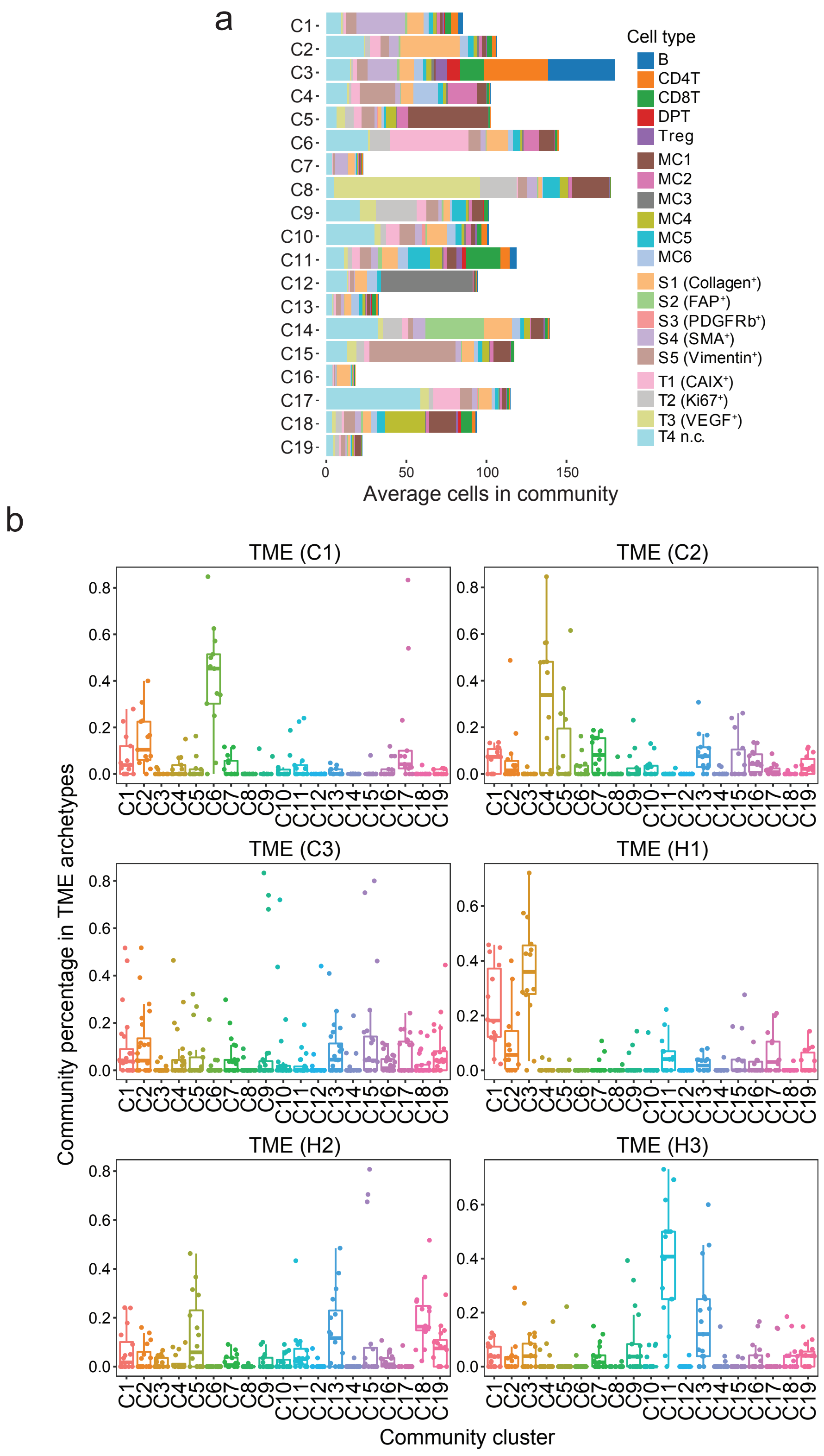

Supplementary Figure 8: Community analysis. **(a)** Microenvironment communities clustered by Phenograph based on the absolute percentage of cells per community, visualized on stacked bar plots indicating the average number of cells being assigned to each community. **(b)** Boxplots showing the percentage of community cluster in each tumor microenvironment (TME) archetype, colored by community cluster. Each boxplot is shown with the median (the center line), interquartile range (IQR), and 1.5 times the IQR (whiskers), with outliers exceeding 1.5 times the IQR. Points in the boxplot represent the community cluster percentage of each image.

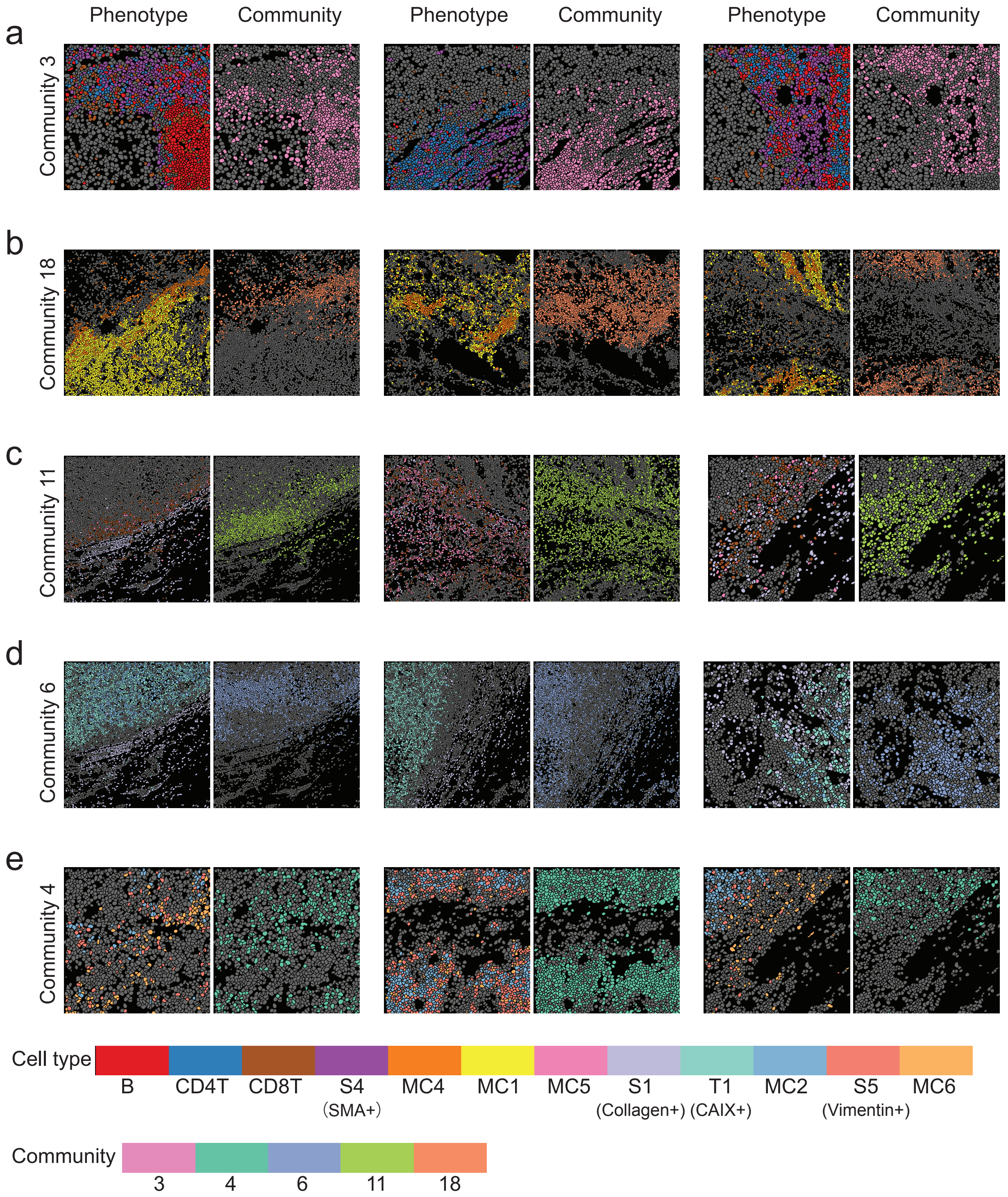

Supplementary Figure 9: Example pseudocolored images of communities. Rows showing three examples of **(a)** community 3, **(b)** community 18, **(c)** community 11, **(d)** community 6, and **(e)** community 4. Columns showing segmented cells colored by cell types (left) and community (right).

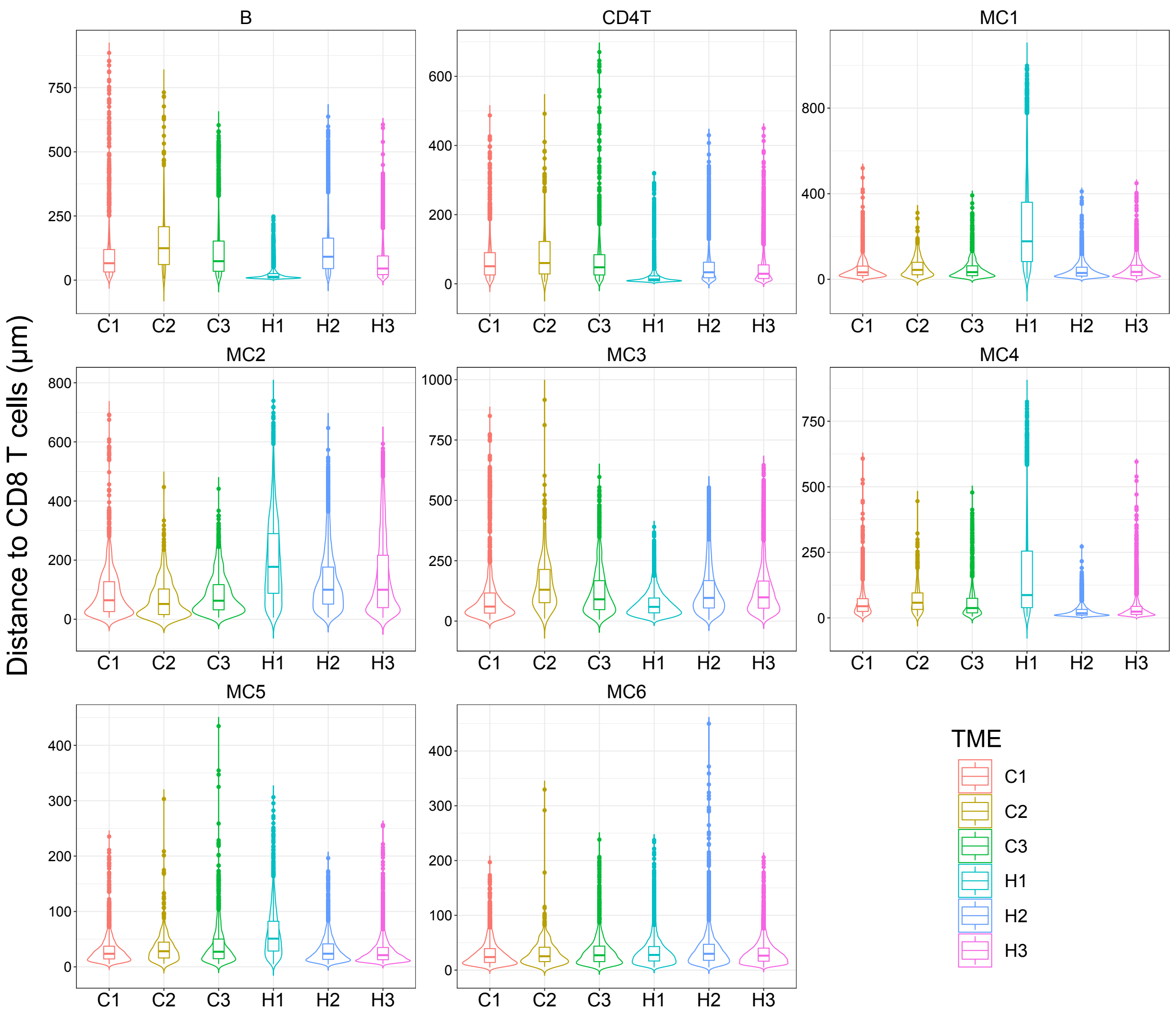

Supplementary Figure 10: The nearest distance between CD8$^+$ T cells and other immune cells in six tumor microenvironment archetypes. Violin plots showing the distribution of the nearest distance of all CD8$^+$ T cells to other immune cells. Boxplots inside violin plots are shown with the median (the center line), interquartile range (IQR), and 1.5 times the IQR (whiskers), with outliers exceeding 1.5 times the IQR.

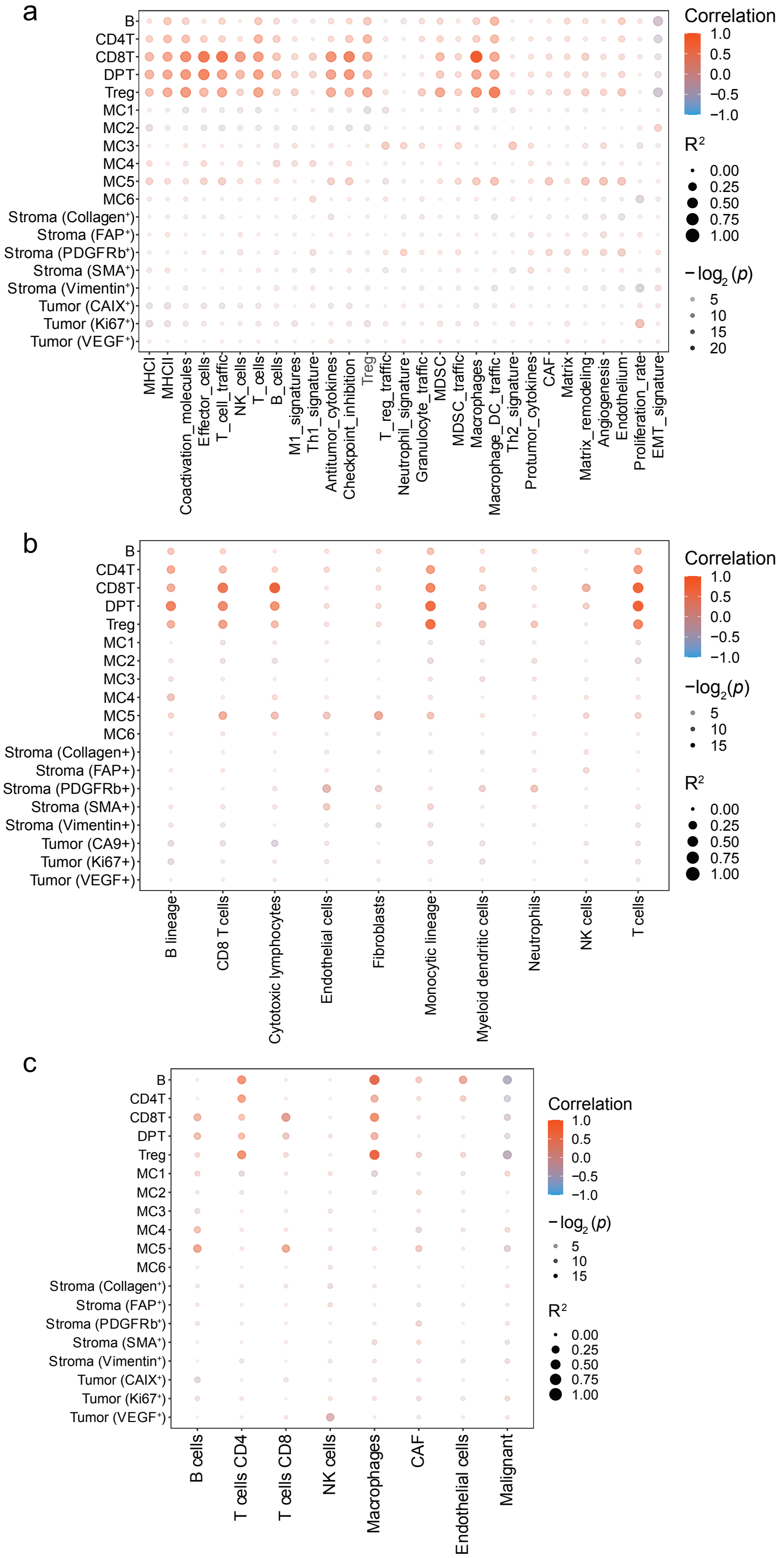

Supplementary Figure 11: The correlation between bulk RNA-seq data and imaging mass cytometry (IMC) data. Spearman's rank correlation between the cell type abundances estimated by averaging over all IMC region of interests from a sample and estimated by **(a)** single-sample gene set enrichment analysis score from 29 curated gene signatures[5], **(b)** MCP-counter[6], and **(c)** CIBERSORTx[7].

References

1. Cui, C. *et al.* Ratio of the interferon-γ signature to the immunosuppression signature predicts anti-PD-1 therapy response in melanoma. *NPJ Genomic Medicine* **6**, 1–12 (2021).

2. Riaz, N. *et al.* Tumor and microenvironment evolution during immunotherapy with nivolumab. *Cell* **171**, 934–949 (2017).

3. Gide, T. N. *et al.* Distinct immune cell populations define response to anti-PD-1 monotherapy and anti-PD-1/anti-CTLA-4 combined therapy. *Cancer Cell* **35**, 238–255 (2019).

4. Liu, D. *et al.* Integrative molecular and clinical modeling of clinical outcomes to PD1 blockade in patients with metastatic melanoma. *Nature Medicine* **25**, 1916–1927 (2019).

5. Bagaev, A. *et al.* Conserved pan-cancer microenvironment subtypes predict response to immunotherapy. *Cancer Cell* **39**, 845–865 (2021).

6. Becht, E. *et al.* Estimating the population abundance of tissue-infiltrating immune and stromal cell populations using gene expression. *Genome Biology* **17**, 218 (2016).

7. Newman, A. M. *et al.* Determining cell type abundance and expression from bulk tissues with digital cytometry. *Nature Biotechnology* **37**, 773–782 (2019).
