# Supplementary material for: Multiplexed imaging mass cytometry reveals distinct tumor-immune microenvironments linked to immunotherapy responses in melanoma
Source: Commun Med (Lond). 2022 Oct 21;2:131. doi: 10.1038/s43856-022-00197-2 (PMC9587266; doi:10.1038/s43856-022-00197-2)
Supplement: Supplementary file 3 — Reporting Summary [file 43856_2022_197_MOESM3_ESM.pdf]

nature portfolio

Corresponding author(s): Wenxian Yang, Yan Kong, Rongshan Yu
Last updated by author(s): Aug 18, 2022

Reporting Summary

Nature Portfolio wishes to improve the reproducibility of the work that we publish. This form provides structure for consistency and transparency in reporting. For further information on Nature Portfolio policies, see our Editorial Policies and the Editorial Policy Checklist.

Statistics

For all statistical analyses, confirm that the following items are present in the figure legend, table legend, main text, or Methods section.

n/a	Confirmed	
☐	☒	The exact sample size (*n*) for each experimental group/condition, given as a discrete number and unit of measurement
☐	☒	A statement on whether measurements were taken from distinct samples or whether the same sample was measured repeatedly
☐	☒	The statistical test(s) used AND whether they are one- or two-sided<br>*Only common tests should be described solely by name; describe more complex techniques in the Methods section.*
☐	☒	A description of all covariates tested
☐	☒	A description of any assumptions or corrections, such as tests of normality and adjustment for multiple comparisons
☐	☒	A full description of the statistical parameters including central tendency (e.g. means) or other basic estimates (e.g. regression coefficient) AND variation (e.g. standard deviation) or associated estimates of uncertainty (e.g. confidence intervals)
☐	☒	For null hypothesis testing, the test statistic (e.g. *F*, *t*, *r*) with confidence intervals, effect sizes, degrees of freedom and *P* value noted<br>*Give P values as exact values whenever suitable.*
☒	☐	For Bayesian analysis, information on the choice of priors and Markov chain Monte Carlo settings
☒	☐	For hierarchical and complex designs, identification of the appropriate level for tests and full reporting of outcomes
☐	☒	Estimates of effect sizes (e.g. Cohen's *d*, Pearson's *r*), indicating how they were calculated

Our web collection on statistics for biologists contains articles on many of the points above.

Software and code

Policy information about availability of computer code

Data collection	IMC data were acquired using a Hyperion Imaging System (Fluidigm). All operations were conducted following manufacturer's procedure. Briefly, images were laser ablated at 200 Hz, and raw data were acquired using a commercial acquisition software (Hyperion Imaging System, Fluidigm). The state of Hyperion Imaging System was monitored by interspersed acquisition of data from tuning slide (Fluidigm).
Data analysis	For IMC data analysis, all images were checked by inspecting all marker staining patterns in the MCD Viewer (Fluidigm, v1.0.560.2). Raw data (.mcd files) were converted to TIFF format using the imctools Python package (https://github.com/BodenmillerGroup/imctools). Then we used in-house developed segmentation tool (https://github.com/xmuyulab/Dice-XMBD) and protein quantification pipeline (will be available upon publication) to perform single cell segmentation and protein quantification for each image. All clustering analyses were performed with the R package FlowSOM (v1.18.0) and Phenograph (v0.99.1). For spatial analysis, the R package NeighbouRhood (v0.3.0) was used for cell-cell interactions/avoidances, and Louvain community detection method (Python package, v0.15) was used for community analysis. Immune cell frequencies of bulk RNA-seq data were inferred using CIBERSORTx (https://cibersortx.stanford.edu/) and MCP-counter (http://134.157.229.105:3838/webMCP/). Differential expression genes (DEGs) were identified using GLM function in the R package edgeR (v3.28.1). Kaplan-Meier analysis and univariable Cox proportional-harzards models were performed using survival (R package, v3.2.3).

For manuscripts utilizing custom algorithms or software that are central to the research but not yet described in published literature, software must be made available to editors and reviewers. We strongly encourage code deposition in a community repository (e.g. GitHub). See the Nature Portfolio guidelines for submitting code & software for further information.

Data

Policy information about availability of data

All manuscripts must include a data availability statement. This statement should provide the following information, where applicable:

- Accession codes, unique identifiers, or web links for publicly available datasets
- A description of any restrictions on data availability
- For clinical datasets or third party data, please ensure that the statement adheres to our policy

Raw IMC images and processed data, including source data for the figures, are deposited in Zenodo with the identifier [https://doi.org/10.5281/zenodo.6838169]. Previously published melanoma RNA-seq datasets reanalyzed here are referenced to and available accordingly. All other data are available from the corresponding author on reasonable request.

Human research participants

Policy information about studies involving human research participants and Sex and Gender in Research.

Reporting on sex and gender	NA
Population characteristics	Patients clinicopathologic characteristics were summarized in Supplementary Table 1 and Supplementary Table 2.
Recruitment	All tumor tissue samples were obtained from melanoma patients with anti-PD-1 monotherapy at Peking University Cancer Hospital, Beijing, China. Patients were treated between March 2016 and March 2019.
Ethics oversight	Medical Ethics Committee of the Peking University Cancer Hospital and Institute (2019KT92).

Note that full information on the approval of the study protocol must also be provided in the manuscript.

Field-specific reporting

Please select the one below that is the best fit for your research. If you are not sure, read the appropriate sections before making your selection.

☒ Life sciences ☐ Behavioural & social sciences ☐ Ecological, evolutionary & environmental sciences

For a reference copy of the document with all sections, see nature.com/documents/nr-reporting-summary-flat.pdf

Life sciences study design

All studies must disclose on these points even when the disclosure is negative.

Sample size	No statistical method was used to predetermine sample size and sample selection of this study was based on sample availability.
Data exclusions	Twenty-nine tissue samples were excluded as they did not meet the IMC experimental requirement.
Replication	Analysis of observational data and no further replication analyses.
Randomization	Randomization not relevant to the analysis of observational data.
Blinding	All data acquisition was conducted blinded to corresponding clinical data.

Reporting for specific materials, systems and methods

We require information from authors about some types of materials, experimental systems and methods used in many studies. Here, indicate whether each material, system or method listed is relevant to your study. If you are not sure if a list item applies to your research, read the appropriate section before selecting a response.

Materials & experimental systems

n/a	Involved in the study	
☐	☒	Antibodies
☒	☐	Eukaryotic cell lines
☒	☐	Palaeontology and archaeology
☒	☐	Animals and other organisms
☐	☒	Clinical data
☒	☐	Dual use research of concern

Methods

n/a	Involved in the study	
☒	☐	ChIP-seq
☒	☐	Flow cytometry
☒	☐	MRI-based neuroimaging

Antibodies

Antibodies used

All Antibodies used in this study are described in Supplementary Table 4.

Validation

In total of 35 antibodies, 25 labelled antibodies were purchased from Fluidigm(https://www.fluidigm.com) and the remaining 10 unlabelled antibodies were purchased from Abcam (https://www.abcam.com/). Antibodies from Abcam were conjugated with metals using MaxparX8 Multimetal Labeling Kit (Fluidigm, 201300) following the manufacturer's protocol. All conjugated antibody titration and specificity were tested by visual comparison of IMC images of tissue slides from melanoma patients.

Clinical data

Policy information about clinical studies

All manuscripts should comply with the ICMJE guidelines for publication of clinical research and a completed CONSORT checklist must be included with all submissions.

Clinical trial registration

The study is not a clinical trial.

Study protocol

NA

Data collection

All clinical data were obtained from the pathology records of patients.

Outcomes

No specific outcome in this study.
